# Personality and predictability in farmed calves using movement and space-use behaviours quantified by ultra-wideband sensors

**DOI:** 10.1098/rsos.212019

**Published:** 2022-06-08

**Authors:** Francesca Occhiuto, Jorge A. Vázquez-Diosdado, Charles Carslake, Jasmeet Kaler

**Affiliations:** School of Veterinary Medicine and Science, University of Nottingham, Sutton Bonington Campus, Leicestershire LE12 5RD, UK

**Keywords:** animal personality, predictability, repeatability, inter-individual variability, movement, space use

## Abstract

Individuals within a population often show consistent between individual differences in their average behavioural expression (personality), and consistent differences in their within individual variability of behaviour around the mean (predictability). Where correlations between different personality traits and/or the predictability of traits exist, these represent behavioural or predictability syndromes. In wild populations, behavioural syndromes have consequences for individuals' survival and reproduction and affect the structure and functioning of groups and populations. The consequences of behavioural syndromes for farm animals are less well explored, partly due to the challenges in quantifying behaviour of many individuals across time and context in a farm setting. Here, we use ultra-wideband location sensors to provide precise measures of movement and space use for 60 calves over 40–48 days. We are the first livestock study to demonstrate consistent within and between individual variation in movement and space use with repeatability values of up to 0.80 and CVp values up to 0.49. Our results show correlations in personality and predictability, indicating the existence of ‘exploratory’ and ‘active’ personality traits in farmed calves. We consider the consequences of such individual variability for cattle behaviour and welfare and how such data may be used to inform management decisions in farm animals.

## 1. Introduction

Variation in individual behaviour has been observed in several animal species [1,2]. In wild animal populations, such differences in behaviour affect individuals' space and resource use [3], as well as within and between species interactions [4] with implications for disease susceptibility [5]. In farm animals, these differences can impact animal welfare and productivity [6,7], and identifying changes in an individual's mean behaviour (personality) or changes in the behavioural variation within individuals (predictability) can be used as early warning for disease as they precede or accompany subclinical/clinical signs [8,9] and as indicators for the welfare status of the animals [10–12]. However, in cases where each individual has a different baseline, targeting the expression of behaviours that vary from the population mean as potential sick or stressed animals can lead to mislabelling. Therefore, measuring between and within individual variability in farm animal behaviour and understanding what behaviours show variability or consistency and under what circumstances is extremely important in moving towards individualized health and welfare planning.

The variation in animal behaviour within a population can be divided into different levels. We define the average behavioural expression of an individual as ‘behavioural type’, and when the differences in behavioural types between individuals are consistent, they are commonly called animal personality traits [13,14]. Animal personality is often quantified by the measure called repeatability, which is computed by extracting the amount of variation attributed to the individual and dividing it by the total variation [2]. Therefore, a higher repeatability value for a behaviour indicates a higher proportion of variation being due to differences among individuals in that behaviour. After the inter-individual component of the variation is accounted for there can be a residual amount of variation that remains, which is called residual intra-individual variability (rIIV) or predictability [15,16]. In addition, these types of variation can be correlated at the within individual level: different behavioural types can be correlated with each other, giving rise to behavioural syndromes [17], and predictability of different behaviours can be correlated with each other creating predictability syndromes [18]. Behavioural types can also be associated with higher or lower levels of predictability, which would indicate the importance of measuring predictability as an additional characteristic of animal behaviour, potentially implicated in fitness [19].

In farm animals, the personality of calves has received a lot of recent focus [20–23], with evidence that calves show repeatable inter-individual differences in behaviours that have been labelled as the personality traits ‘fearfulness’ and ‘sociability’ [21,24]. However, these studies research personality by using standardized assays, where individuals' responses to novel environments are repeatedly tested. As such, animal personality research in a farm setting conducted to date may not be widely generalizable [25], and is still lacking any analysis and quantification of rIIV. In recent years precision livestock farming—the use of sensors to gather data on the animal or in its environment—has allowed monitoring of large numbers of animals in detail and for extended periods of time [12,26–28]. This type of data provides the granularity required for the analysis of inter- and intra-individual variability in behaviour and provides the opportunity to achieve a reliable and objective measures of animal personality and predictability [19,29]. For example, measures of movement and space use show inter- and intra-individual variation in both wild and captive animals [30–32], and such data can easily be collected by modern sensor technologies in farms. Movement behaviours are intrinsically linked with many classically defined personality traits such as ‘exploration’, which is defined by the reaction of an individual towards new environments or objects [14,31,33], and ‘activity’, which corresponds to a measure of the amount of movement performed by an individual [14].

Here, we use precision livestock technologies to measure individual movement patterns as they occur under normal management and detect variation at the between and within individual level to (i) investigate whether calves display different personality types by measuring the individual differences and temporal consistency (repeatability) of measures of movement and space use—total distance travelled, core area used, total area used, site fidelity and time spent in the feeder area; (ii) investigate potential differences in predictability; (iii) investigate the correlation between these variables to uncover the presence of behavioural syndromes, and the correlation between personality and predictability of the different measures.

## 2. Material and methods

### 2.1. Animals, housing and farm management

**Figure 1.**
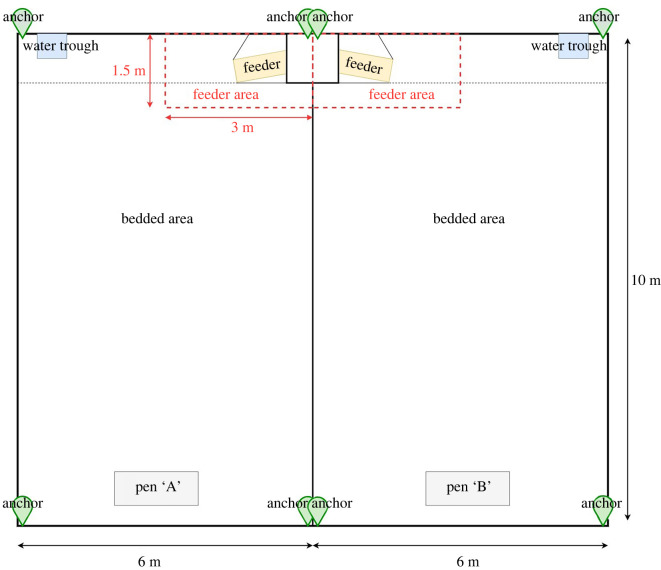
Diagram of the pen indicating the position of the anchors, the feeder and the extent of the ‘feeder area’.

The study took place at the Centre for Dairy Science Innovation at the University of Nottingham, UK, between October 2020 and April 2021. Sixty calves were divided into four cohorts of 14–16 calves each, enrolled between October 2020 and February 2021. The four cohorts comprised a total of 60 calves, of which 36 were Holstein Friesian dairy heifers, seven Holstein Friesian bulls and 17 cross breed calves, of which eight were crossed with Aberdeen Angus and eight with Belgian Blue as described in table 1. At birth, the calves were housed in pairs as per regular farm management until a minimum of 14 calves at least two weeks old was reached to form a cohort. Each cohort was then moved as a group to one of the two adjacent straw-bedded 6 m × 10 m pens (figure 1) where they stayed for 7–10 weeks. The calves were fed milk replacer from an automatic feeder with an initial entitlement of 10 l, allocated to each calf through RFID recognition. They also had ad-lib access to concentrates, chopped straw and water. The calves in the first two cohorts were on an individualized feeding plan based on their age, following normal farm practice, and received a gradually reducing amount of milk until they reached weaning age. The calves in the last two cohorts were all placed on the same plan and the milk allowance was decreased at the same time until they were all fully weaned by day 58. Cohorts 1, 3 and 4 were monitored for 48 days, while cohort 2 was monitored for 40 days due to operational requirements of the farm. All methods were carried out in accordance with relevant guidelines and regulations. Ethical permission for all the observational procedures was obtained for the School of Veterinary Medicine and Science, University of Nottingham (unique reference no. 1481 150603).

**Table 1.** Number of calves in each cohort divided by sex and breed: Holstein Friesian (HF), Holstein Friesian × Belgian Blue (HF × BB) and Holstein Friesian × Aberdeen Angus (HF × AA).

cohort	*N*	sex		breed			age (mean at day 0)	Pen
		F	M	HO	HO × BB	HO × AA		
1	14	12	2	13	0	1	38.7	A
2	14	12	2	10	3	1	41.5	B
3	16	14	2	10	3	3	24.1	B
4	16	13	3	10	2	4	31.6	A

### 2.2. Sensor data collection

**Figure 2.**
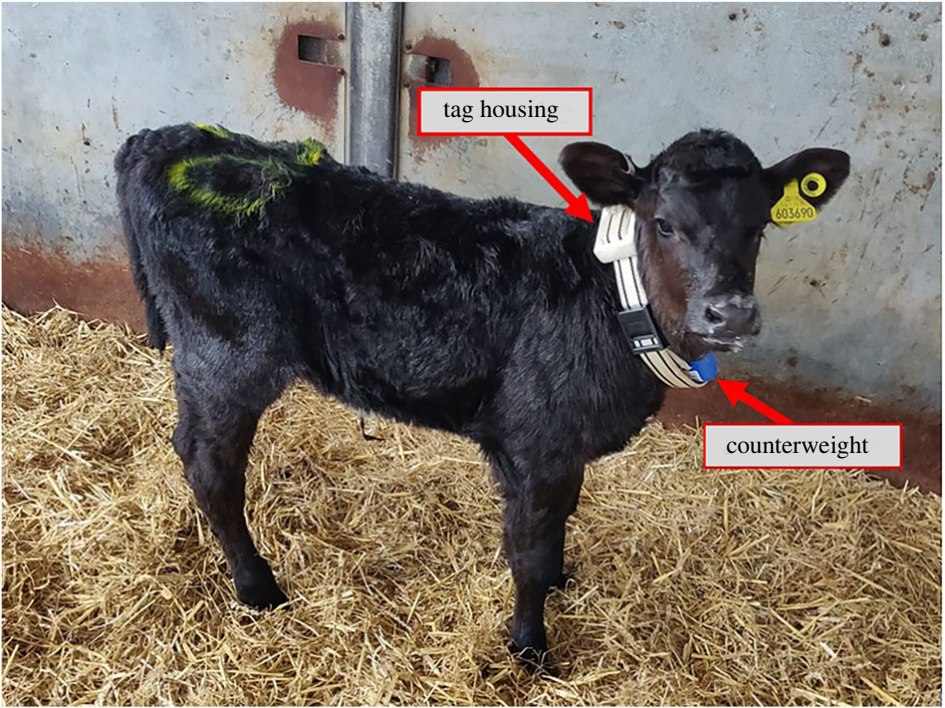
Picture of one of the calves enrolled in the trail wearing a collar to illustrate the position of the tag housing (on the top of the collar) and of the counterweight (at the bottom of the collar).

The location data were collected using Sewio Leonardo iMU tags, which use ultra-wideband [34] technology to report the relative local coordinates (*x*, *y*) of the individual and were set to a frequency of 1 Hz. The tags were mounted on a collar worn by the calves and recorded the location of all the individuals continuously for the whole duration of the trial. Counterweights in the neck collars were used to help maintain sensors in the same position at the collar (figure 2). To minimize any effect of wearing the collars on the behaviour of the calves they were fitted two to three weeks prior to their move to the group pen, which allowed habituation to the novelty, and data from the first day in the group pen was discarded in order to account for the initial adjustment to the new environment.

The location position system uses four anchors in order to determine the location via triangulation. Figure 1 shows a schematic diagram of all the different zones for the two different pens. The feeder zones consist in an area of 1.5 × 3 m around the automatic feeder as shown in figure 1.

Before the start of the trails, the location data were validated using 20 sensors at nine static positions in the pen in a similar manner to [9] using the mean circular error probability (CEP) and a measure of the accuracy calculated as the mean distance between the known ground truth location and the sensor derived location (DIST). The CEP is used as a measure of precision of the location sensors, representing the radius of a circle, centred at the mean location, where 50% of the location points lie. For the selected locations in the pen, the mean CEP was 0.15 m (range 0.12–0.28 m). DIST was found to be 0.17 m (range = 0.13–0.33 m).

Daily summaries of ambient temperature were collected from temperature sensors located in the building (ALTA 900 MHz Industrial Humidity Sensors with Probes).

### 2.3. Pre-processing and cleaning of positional data

Any times when calf behaviour might have been affected by human proximity were removed from the dataset and there were some short losses of data due to power cuts and battery drainage of the tags. Overall, 2 115 131 h of the total 66 432 calf-hours (representing 3.14% of the data) for all four cohorts were removed. Location points outside the pen coordinates were also removed from the original raw data. Finally, in order to improve precision, raw position data were smoothed using a simple moving average over a 10 s moving window. Smoothed location data were used to directly compute movement and space use behaviours.

### 2.4. Movement and space use behaviours

For each individual calf, the total time spent in the trial was divided into four-day observational time units and for each of these we computed the following movement and space use behaviours: total distance travelled (m), ‘total’ area used (m^2^), ‘core’ area used (m^2^), site fidelity and total time spent in the feeder area (min). The total distance travelled was computed as the sum of the total distance travelled over four consecutive days. The area used was measured based on the utility distribution (UD), which represents the probability distribution of the animal's use of the space available: in animal space use analysis, the ‘core’ and ‘total’ area are used to represent the area that corresponds to the cumulative 50% and 95% of the observed density on the UD [35,36]. Both the core and total area used were computed using adehabitat package in *R* using location processed data over four consecutive days [37]. Site fidelity represents the level of overlap or similarity of UDs at different time points and was computed using the average of Hellinger distance over all possible combinations of pairs of days within the observational time unit [35,38]. Total time spent in the feeder area represents the overall time spent within the feeder area for the observational time unit and was computed by counting the number of points that lie within the area previously defined (figure 1). Because the location was collected at a 1 Hz frequency the number of points is equal to the number of seconds spent in the area. These five behaviours were selected because they are the most commonly studied movement behaviours in animal personality studies [31,32,39,40], and because in our view they each capture a different and interesting behavioural aspect of individuals' movement and space use. The length of the time units was chosen to have sufficient temporal resolution while also reducing autocorrelation [41] and enabling the computation of site fidelity by comparing multiple data points over a time period.

### 2.5. Statistical analysis

All statistical analyses were carried out using R software v. 3.5.1 [42] and the code was adapted from Hertel *et al*. [31]. To detect differences between individuals we fitted a mixed effect linear model using the lme4 package in R [43] for each of the behaviours defined above:

2.1 $$Y_{ij}=(\beta_{0}+{\mathrm{ind}}_{0j})+\beta_{1}X_{1ij}+\cdots +\beta_{8}X_{8ij}+e_{0ij},$$

where $Y_{ij}$ represents one of the movement and space use behaviours, *i* represents a repeated measure of *Y* for each observational time unit, *j* represents an individual animal, *β*_0_ is the mean value of average individual responses and ind_0*j*_ is the individual contribution estimated as the random intercept [44]. $\beta_{1}X_{1ij},\;\beta_{2}X_{2ij},\ldots ,\;\beta_{8}X_{8ij}$ represent the vector coefficients for the four-day observational time unit, cohort, age of the calf (taken at the end of the observational time unit), average temperature in the pen, breed, sex, weaning stage and health status of the calf, respectively. The interaction between the effects of cohort and observational time unit was added to account for the fact that the cohorts were recruited at different times of the year and might experience different undetected environmental gradients. The random intercept is assumed to be normally distributed (*N*) with zero mean and variance ($\Omega_{\mathrm{ind}}$), representing the *between individual* variance ($V_{{\mathrm{ind}}_{0}}$). The residual error $e_{0ij}$ is assumed to be normally distributed with zero mean and variance ($\Omega_{e}$) representing the *within individual* variance ($V_{\mathrm{eo}}$) [44]. Weaning was coded as a categorical variable with three levels: not weaning, step-down stage (when the calves were entitled to a reduced amount of milk) and fully weaned. The health status was determined based on whether the calves had received treatment from the farm staff: the calf was considered ‘sick’ when the day and/or the previous two days of the treatment administration fell within the observational time unit, while it was considered ‘convalescent’ when the three days following the treatment fall within the observational time unit.

The models allowed us to calculate the adjusted repeatability (*R*) for each behaviour across the whole trial, by extracting the different levels of variation and dividing the variation due to the random effect ($V_{{\mathrm{ind}}_{0}}$) by the total phenotypic variation $\left(V_{{\mathrm{ind}}_{0}}+V_{\mathrm{eo}}\right)$ [2,31,44–46]:

2.2 $$R=\frac{V_{{\mathrm{ind}}_{0}}}{(V_{{\mathrm{ind}}_{0}}+V_{\mathrm{eo}})\;}.$$

To quantify predictability of the different movement and space use behaviours as well as the correlation between these measures, we adopted a Bayesian approach and fitted univariate double hierarchical generalized linear model (DHGLM) [47,48] to each of the dependent variables used above using the brms package in R [49]. The model can be written as equations (2.3)–(2.4) for mean and equations (2.5)–(2.8) for the dispersion part of the models [45].

2.3 Y=Xβ+Zα+ε,

2.4 $$\alpha ∼N(0,I_{m}\sigma_{\alpha }^{2}),$$

2.5 $$\varepsilon ∼N(0,\mathrm{Diag}\{\sigma_{\varepsilon }^{2}\}),$$

2.6 $$\log(\sigma_{\varepsilon })=\eta_{d},$$

2.7 $$\eta_{d}=\;X_{d}\beta_{d}+Z_{d}\alpha_{d}$$

2.8 $$\;\mathrm{and}\;\alpha_{d}∼N\left(0,I_{m}\omega_{\alpha_{d}}^{2}\right).$$

The model described above allows us to include fixed and random effects for the dispersion part of the model. In the model, α represents individual-specific random effect variation, *X* represents the fixed effects, *Z* represents the random effects and ε represents the residual deviations from the prediction. Terms $X_{d}\;$ represent the fixed effects for the dispersion part of the model, $Z_{d}\alpha_{d\;}$ represents the random effect component of the dispersion and $\omega_{\sigma }^{2}$ represents the dispersion model hyperparameter. Between individual random effect of variance (α) is assumed normally distributed as well as $\alpha_{d\;}$ and individual-specific standard deviations are assumed to follow a lognormal distribution. For both the mean and the dispersion parts of the model we use the same fixed and random effects as described in equation (2.1).

We ran the models with uninformative priors for four chains, 10 000 iterations using Markov chain Monte Carlo (MCMC) sampling, with a burn-in of 5000 and a thinning interval of 10. A Raftery–Lewis diagnostic test was run to ensure that the iterations exceeded the minimum requirement and mixing of the chains was assessed using the R-hat convergence diagnostic [50,51]. From these models we were able to calculate the coefficient of variation in predictability (CV_p_), which represents the standardized population-level measure of the degree of variation in predictability between the individuals, with a higher CV_p_ indicating a larger variation in predictability between individuals [45]:

2.9 $${\mathrm{CV}}_{p}=\sqrt{\left(\exp(\omega^{2}{}_{\mathrm{ID}})-1\right)}.$$

Lastly, we fitted a multivariate DHGLM, which included all the dependent variables from the previous models, which were scaled in this case, and obtained the correlations between these variables at the inter- and intra-individual level. We ran the models with uninformative priors and ran the model for four chains, 10 000 MCMC iterations, with a burn-in of 6000 and a thinning interval of 4. Diagnostics tests were run as for the previous model.

## 3. Results

### 3.1. Repeatability

**Figure 3.**
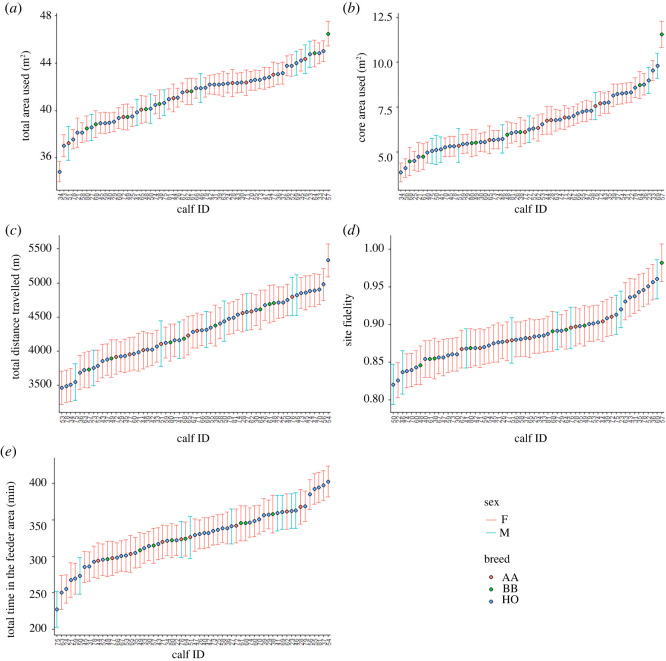
Behavioural types of individual calves for each of the target behaviours: total area used (*a*), core area used (*b*), total distance travelled (*c*), site fidelity (*d*) and total time spent in the feeder area (*e*). Estimates are based on mixed effect linear models after controlling for observational time unit, cohort, age, temperature, breed, sex, weaning stage and health status. The error bars represent the standard deviation.

All measures of movement and space use were repeatable, indicating consistent inter-individual differences, or personality traits in the calves. However, individual identity explained different total variation in our models (electronic supplementary material, table S1). The highest repeatability was for the core area used (*R* = 0.80 [0.77, 0.83], *N* = 60) and total area used (*R* = 0.78 [0.74, 0.81]). Total distance travelled had moderate repeatability (*R* = 0.35 [0.30, 0.40]) as did total time spent in the feeder area (*R* = 0.33 [0.28, 0.38]), while site fidelity had low repeatability (*R* = 0.22 [0.18, 0.27]). The individual behavioural types are shown in figure 3.

### 3.2. Predictability

**Figure 4.**
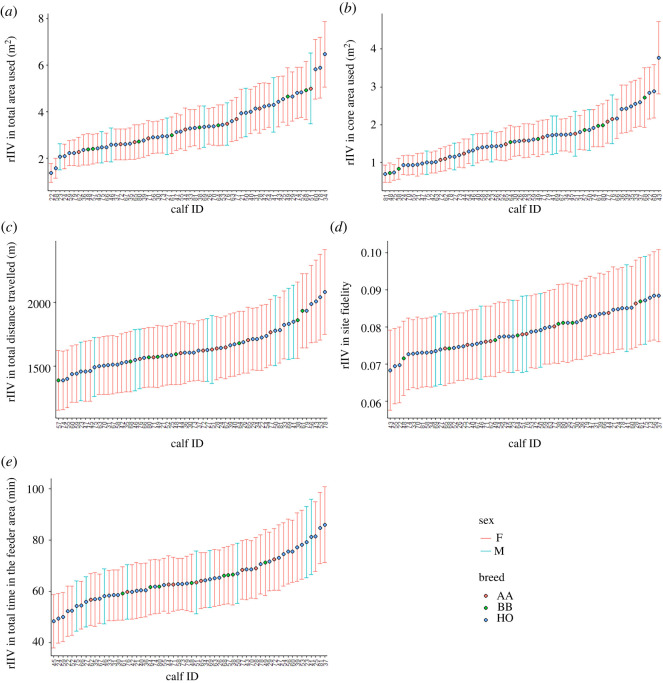
Residual intra-individual variation (rIIV) of individual calves for each of the target behaviours: total area used (*a*), core area used (*b*), total distance travelled (*c*), site fidelity (*d*) and total time spent in the feeder area (*e*). Estimates are based on double hierarchical mixed models after controlling for age, day number and cohort. The ridges indicate the posterior 95% credible interval.

Calves showed consistent differences in rIIV, indicating variation in predictability between the calves in this study (figure 4). Again, the highest variation in predictability was for the core area used (CVp = 0.49 [0.35, 0.62], *N* = 60) and total area used (CVp = 0.42 [0.30, 0.55]). This measure was low for all the other variables (total distance travelled: CVp = 0.18 [0.07, 0.27]; total time spent in the feeder area: CVp = 0.22 [0.13, 0.34]; site fidelity CVp = 0.14 [0.013, 0.24]).

### 3.3. Correlation between behavioural type and predictability

**Figure 5.**
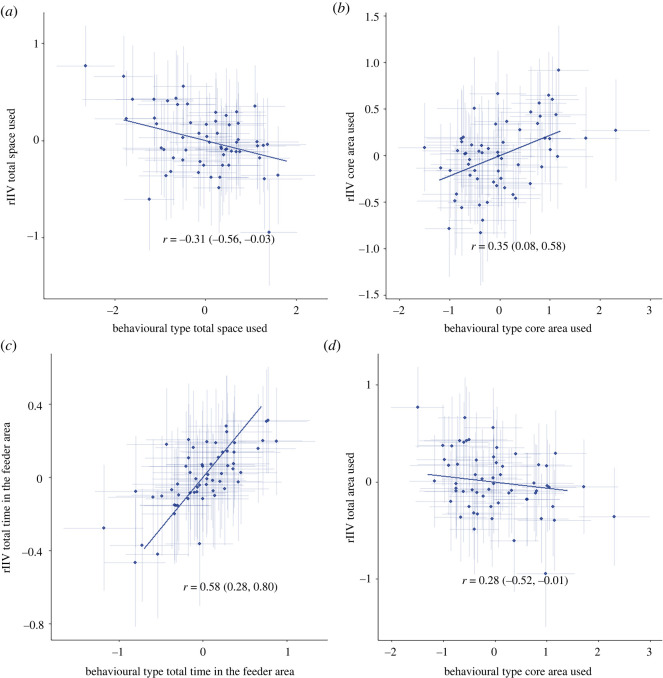
Among individual correlation between behavioural type and residual intra-individual variation (rIIV) of total area used (*a*), core area used (*b*), total time spent in the feeder area (*c*) and between the behavioural type of core area used and the rIIV of total area used (*d*). The error bars indicate the posterior 95% credible interval.

Several of the variables had a correlation between behavioural type and rIIV of the same variable (figure 5). Since a larger amount of rIIV corresponds to more unpredictable behaviour, a positive correlation between behavioural type and rIIV is a negative correlation with predictability and vice versa. The individual behavioural type for total area used was negatively correlated with the rIIV (cor = −0.31 [−0.56; −0.03]), meaning that individuals with a larger total area used were more predictable in the size of the area they used. By contrast the behavioural type for core area used was positively correlated with the rIIV (cor = 0.35 [0.08; 0.58]), indicating that the individuals with larger core area used were more unpredictable. A positive correlation between behavioural type and rIIV existed also for the time spent in the feeder area (cor = 0.58 [0.28; 0.80]), which indicated that the individuals that spent more total time in the feeder area were more unpredictable. There is also a negative correlation between the behavioural type of the core area used and the rIIV of the total area used (cor = −0.28 [−0.52; −0.01]), meaning that individuals with larger core area used were more predictable in their total area used.

### 3.4. Behavioural syndromes

**Figure 6.**
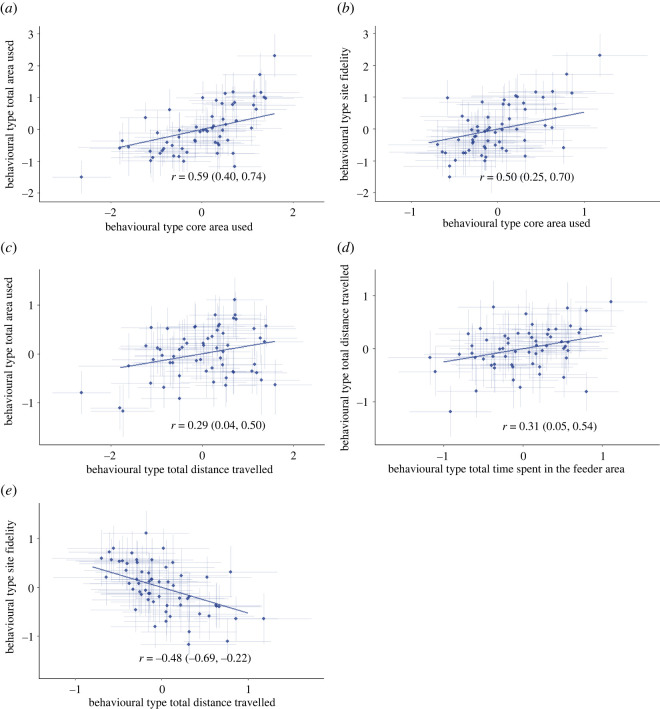
Among individual correlation between behavioural types: total area used and core area used (*a*), site fidelity and core area used (*b*), total area used and total distance travelled (*c*), total distance travelled and total time spent in the feeder area (*d*) and site fidelity and total distance travelled (*e*). The error bars indicate the posterior 95% credible interval.

There were also several correlations between different behavioural types, indicating the existence of behavioural syndromes (figure 6). As we expected total area used and core area used were positively correlated (cor = 0.59 [0.40; 0.74]), and core area used was positively correlated with site fidelity (cor = 0.50 [0.25; 0.70]). Total distance travelled was positively correlated with total area used (cor = 0.29 [0.04; 0.50]) and with time spent in the feeder area (cor = 0.31 [0.05; 0.54]) and negatively with site fidelity (cor = −0.48 [−0.69; −0.22]).

### 3.5. Predictability syndrome

**Figure 7.**
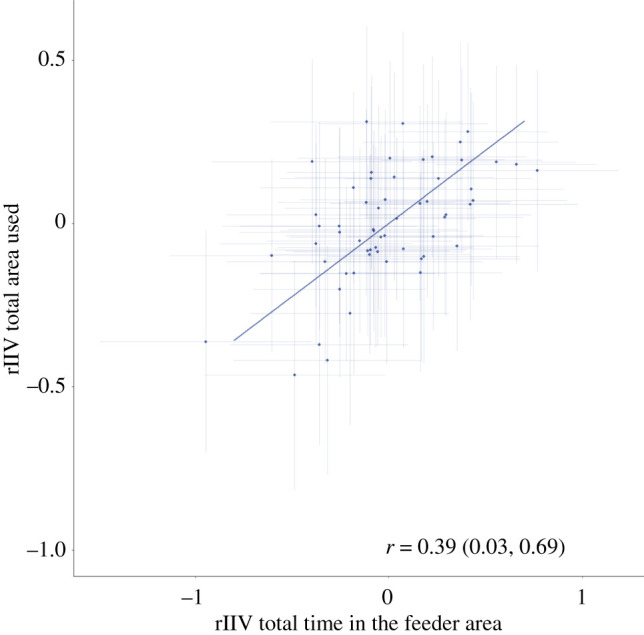
Among individual correlation between the residual intra-individual variability (rIIV) of total area used and the rIIV of the time spent in the feeder area. The error bars indicate the posterior 95% credible interval.

Lastly, there was a correlation between the rIIV of total area used and the rIIV of the time spent in the feeder area (cor = 0.39 [0.03; 0.69]) (figure 7). This indicated the presence of a predictability syndrome in our data.

## 4. Discussion

This is the first livestock study to collect detailed and extensive data on the space use and movement of farm-housed calves using precision technologies and among the first to quantify individual variation in these behaviours in animals, especially at the intra-individual level and the correlations at the inter- and intra-individual level. This quantification of inter- and intra-individual variability adds significant advancement in the field for farmed livestock behaviour and animal personality by adding empirical evidence of heterogeneity between calves at various levels and demonstrating temporal stability for these traits. The results showed that calves display consistent between individual differences in behavioural expression (measured by the level of repeatability) in all the behaviours that we measured: total distance travelled, total area used, core area used, site fidelity and time spent in the feeder area. This evidence of inter-individual differences alongside the presence of temporal stability in movement and space use behaviours indicates that these behaviours can be used to detect potential personality types [52]. In addition, our study indicated consistent between individual differences in the level of rIIV in the behaviours measured, and correlations between behavioural types and rIIV which further supports how these could be operationalized to define personality in calves. Finally, our study demonstrates usefulness of livestock technologies such as location sensors, which allow non-invasive measures of behaviour under normal management for identifying behaviours that show consistent inter and intra-individual differences, rather than having to rely on performing behavioural tests.

The highest value for repeatability was observed in the two space use measures, indicating that the individuals in our study showed consistent within individual differences in the size of their total and core area used. This suggests the presence of an ‘exploratory’ personality trait in calves, as this trait has been defined by the propensity of an individual to inspect new environments or objects [14,31,33]. Therefore, we can speculate that the calves that had a larger space used were more likely to explore the whole of their home pen and investigate new stimuli, such as noises and smells from farm activities around the pen, which would make them more exploratory. The fact that in this study total distance travelled showed a moderate level of repeatability can also indicate the existence of an ‘active’ personality trait in calves, as some individuals consistently walked longer distances than others, which fits with the definition of this trait [14]. A study that tracked the movement of wild brown bears also reported repeatability values that were comparable to our study, with 0.28 repeatability for travel distance, which they considered to be a measure of activity, and 0.26 for displacement, which they considered to be a measure of exploration [30].

Our finding that individuals show consistent variation in their level of predictability highlights the importance of this rarely explored area of animal behaviour. The behaviour with the highest variation in predictability was core area used, followed by total area used. For decades predictability has been considered a part of the human personality framework since it was discovered that individuals display distinct patterns of intra-individual variation in behaviours [29,53]. Our results add to the existing evidence that predictability is a characteristic of animal behaviour [18,19,28]. On a biological level, the variation in predictability can affect the fitness of the individuals in different ways [19]. There are examples in the literature of positive and negative correlations between predictability and health or reproductive success [54,55], however, more research is needed to understand exactly what the correlation could be in calves as our study did not include measures of fitness. For the purposes of farm animal management, it is possible to speculate that unpredictable animals might be less affected by change in the environment and therefore more resilient, while animals that rely on a more consistent pattern of behaviour might struggle to cope with changes.

The correlations between behavioural type and predictability of the observed behaviours further support the importance of measuring predictability. In particular, the positive correlation between the behavioural type and the rIIV of core area used is indicative of higher predictability in calves that prefer to spend most of their time within a smaller area, while individuals that were more unpredictable in this behavioural type, and that on average have a larger core area used, are perhaps more motivated to explore different stimuli when these are presented. Therefore, if the external stimuli are not consistent, as might be expected in a farm environment, on the days when a novel situation is presented the more exploratory calves might be prone to investigate and spend more time near the source of the stimulus, increasing the core area used of those individuals for those days. Several studies have suggested that individuals vary in their response to external stimuli [19,56], but further studies are required to determine whether this is the cause of the observed variation in predictability and its correlation with the size of the space used.

It is possible that the positive correlation between the behavioural type and the rIIV of time spent in the feeder area could be driven by a minimum required time to fulfil the nutritional needs. The calves that spent the least amount of time near the feeder might have only visited the area for the time needed to drink their daily entitlement of milk, and therefore were more predictable, while the ones that spent more time might have been driven by an exploratory tendency. Additionally, there could be an opportunistic motivation to this behavioural pattern: more exploratory individuals might attempt to access the feeder more often depending on external circumstances, such as the identity of the other individuals present in the feeder area, and might occasionally attempt to displace weaker calves. This could indicate resourcefulness and resilience to changes and be beneficial for the individual [57].

The correlations that we observed in our data between behavioural types support the idea of behavioural syndromes, and that there are underlying traits that drive the expression of more than one behaviour [17,18,58]. In our case total distance travelled was positively correlated to total area used and negatively with site fidelity, indicating that the calves that walked longer distances, and therefore were more active, were also exploratory and less likely to return to a favourite spot within the pen. This is consistent with the results reported by Hertel *et al*. [30], where the travel distance was positively correlated with displacement. In our study total distance travelled was also positively correlated with total time spent in the feeder area, and we know from the discussion of this result in the paragraph above that calves that spend more time in this area tend to be the more unpredictable and opportunistic ones, suggesting a possible link between activity and opportunistic behaviour. In addition, we observed a positive correlation between the rIIV of total area used and the rIIV of the time spent in the feeder area. This is one of the first examples of a predictability syndrome in animals [18], supporting the idea of predictability being a trait of the individual in its own right, which can be expressed through different behaviours [16].

Understanding why different personality types exist within a population is complex and beyond the scope of this paper. However, we can rely on well-established ideas such as the pace-of-life syndrome (POLS) hypothesis [59] to understand why groups of behaviours might be correlated at the intra-individual level. This hypothesis posits the existence of a trade-off between maximizing reproduction at the expense of survival (fast strategy) and maximizing survival at the expense of reproduction (slow strategy). As individuals sit on the axis between slow and fast strategy, their expression of different behaviours will reflect their strategy and therefore be correlated with each other at the intra-individual level. This would explain our results as the individuals that walk the furthest, have highest total area used, lowest site fidelity and highest total time spent in the feeder area would correspond to those adopting a ‘fast’ strategy, while the ones that walk smaller distances, occupy a smaller total area used, have high site fidelity and spend less time near the feeder adopt the ‘slow’ strategy.

Our statistical method has avoided the common mistake of using several repeated measures collected from personality tests and combining them using data-reduction techniques, such as principal components analysis (PCA) [22,24,60,61]. Performing a PCA on repeated measures is a violation of the assumptions of this statistical method in terms of observation independence and assuming correlation structures that do not differ among versus within individuals and therefore the use of multivariate generalised linear mixed-models (GLMM) is preferred [46,62]. It must be noted that although we controlled for several fixed effects that we believe might have otherwise confounded the results, there might be some other environmental changes that we could not detect or subclinical disease that was not recorded. In addition, while we tested the temporal repeatability of movement and space use behaviours, we did not test whether they are stable across different contexts and therefore further studies are needed to address this. A way to achieve different contexts would be to change the environment that the individuals are experiencing by modifying the home pen size, group structure or introducing enrichment elements.

The behavioural type and predictability levels of different behaviours can affect the response to management of farm animals and therefore needs to be carefully considered accordingly. For example, animals with a very high site fidelity might be more stressed in response to relocation, while individuals with a particularly high total distance travelled and large space used might struggle more than other individuals to cope if confined to a small area. Overall individuals that are more consistent in their behaviour could experience more distress if their environment is changed, while unpredictable individuals might be more resilient. Future research should focus on the relationship between inter- and intra-individual variability and outcomes such as health and productivity, in order to understand their effects. The combination of precision livestock technologies, which can collect high-quality data for large numbers of individuals and long periods of time, combined with the multivariate mixed-effects linear models to analyses these data, has proven extremely effective as a tool to measure behavioural type and predictability in a non-invasive and methodologically sound way and move us closer to an individualized welfare approach.

## Data Availability

The datasets supporting this article is available online [64] and the code to reproduce the data analysis is available in the electronic supplementary material [65].
